# Exclusions

**Published:** 1916-03-11

**Authors:** 


### THE SCHOOL DOCTOR.

### Exclusions.

Most medical inspectors have their own rules with regard to excluding children, and in general the majority err, if they err at all, in over rather than in under excluding. In other words, they take a graver view of certain conditions than is perhaps justified by experience; but, on the other hand, they safeguard both themselves and the schools by such a policy. In regard to infectious diseases, it is now almost universally recognised that all children suffering from an infectious or contagious disease should be excluded from school so long as they are likely to carry or spread infection. With regard to the "contacts" there is as yet no such unanimity of opinion as obtains in regard to children actually infected, and the rules of various education authorities vary. Similarly, opinions with regard to school closure vary. Some inspectors, especially those who regard things from the medical officer of health's point of view, are inclined to close schools far too frequently. This policy, too, has much to recommend itself, for it undoubtedly saves trouble and minimises risk. But it may entail expense to the authority, and it is doubtful if it is such a good prophylactic measure as it is imagined to be. Children excluded from school as contacts invariably play together on the streets or in the public playing grounds, attend Sunday school or scout practices, and in that way disseminate infection. The school as a factor in spreading infection has been somewhat overestimated, but it is still striking enough to see that in the case of so highly infectious a disease as whooping cough the precautions ordinarily suggested in the case of scarlet fever or measles are hardly ever deemed necessary. Yet from a scholastic, no less than a social point of view, whooping cough is undoubtedly one of the most serious of the school diseases. If schools are to be closed because certain of the children are infected with scarlet fever, it seems equally necessary to close them if some of the children have whooping cough.

### Old and New Views.

In dealing with these acute exanthemata the school doctor will act according to his experience and teaching. If he has been trained in the old-fashioned school that looks upon these diseases as most serious factors of school infection, he will deal drastically with all contacts and insist on exclusion and perhaps closure. If, on the other hand, he holds the more modern view that the school is not such a potent factor in causing the dissemination of these diseases, he will be more lenient and exclude only contacts and actually infected children, and hardly ever find it necessary to close a class or a whole school. Local conditions will, of course, largely influence him in dealing with certain schools, and when there is a general epidemic of disease more drastic treatment is amply warranted.

So much for acute infectious diseases. But there remain a class of children who, while not likely to infect other children directly, are yet a source of trouble in the class, often a nuisance. Children suffering from urinary or fæcal incontinence, for example, should be excluded. Similarly, children suffering from any offensive condition, whether with or without discharge, should also be excluded. Such conditions are chronic otitis media, ozæna or atrophic rhinitis, very foul-smelling breath due to pyorrhœa or septic tonsils, impetigo contagiosa or the various forms of tinea, open sores, unsightly skin disease, blepharitis, and purulent conjunctivitis. In most of these conditions there is a strong possibility of the condition being contagious, and as such it amply justifies exclusion. Where this possibility becomes a certainty, as in cases of venereal disease, open tuberculosis, or parasitic disease, and in cases of verminous children, exclusion must be insisted upon; equally so in cases where the diagnosis is not certain, but where there is a strong possibility that the condition is contagious. Much tact is demanded from the school doctor in dealing with some of these doubtful cases, especially when they are under the care of local practitioners who have given certificates to the effect that the child may attend school. In one case a boy of twelve was seen suffering from ulceration of the hard and soft palates; the condition was acute enough to warrant exclusion apart from any other possibility, but it was also probably syphilitic in character, and on that ground the child was promptly excluded. He returned next day with a certificate signed by his home doctor to the effect that the condition was due to "simple inflammation," and that he was fit to attend school, while the medical practitioner wrote to the headmaster expostulating on the gross irregularity committed by the school doctor in "interfering with a case that did not concern him." Fortunately, the headmaster was a tactful individual, who interviewed the parents of the child and induced them to ask for a consultation, when the condition was regarded as something grave enough to justify hospital treatment. As a result the boy was absent from school for several months. Exclusion in this case, whatever the diagnosis might have been—and there was some doubt as to whether the lesion was tubercular or syphilitic—was, therefore, fully justified.

### Valid Causes of Exclusion.

Exclusion is also desirable in cases of tuberculosis of the lungs, in malcompensated heart lesions, in weakly and malnourished children, and generally in all conditions in which the child is incapable of benefiting by the ordinary routine work of the school, or unable to take part in the ordinary play of his fellow-pupils. Cases of advanced paralysis, of grave heart trouble, of severe deformity, for instance, are all unsuitable for the ordinary school; they should be relegated to a special school where proper special attention can be given to the children. In cases of feeble-mindedness provision is now generally made for the reception of such children at special schools, and the same holds with regard to physically defective children. But in cases of malnutrition, bad heart disease, and pulmonary phthisis there is sometimes great difficulty in finding suitable occupation for the children excluded from school. The school doctor should be in close touch with the various voluntary agencies in his district, so as to be able to arrange for the reception of such children in suitable homes of recovery, or to give them the benefit of a country holiday or a change to the seaside. Merely to exclude a tuberculous child from school, not caring what happens to him at home, is an unsound policy. The Care Committee will generally give valuable help in these circumstances. Its visitors should be instructed to call at the child's home and interview the parents, suitably advising them according to the directions given by the school medical inspector. Some authorities—notably in America and in Australia—now publish admirable leaflets on the home treatment of consumptive children and children with malcompensated heart disease.

Finally, there is the abnormal child to consider. Exclusion is not often warranted in his case, except where a physical lesion, such as chorea or epilepsy, is the possible factor in causing his special abnormality. Epileptic children should be promptly excluded. There is no reason for their retention in school; they disturb the classes, are undesirable companions for the other children, and it is very doubtful if they benefit in the least from ordinary class instruction. Their proper place is in a special colony for epileptics. Children with chorea should be excluded if their home conditions are favourable for that mental and physical rest that they need for their complete cure; otherwise it is better to retain them in class and instruct the teacher to deal with them on a sort of preferential basis, giving them something to occupy their time, but not exacting from them any work that demands strain or attention. Other abnormal children must be treated individually, exclusion, in general, is only warranted if the child is a real trouble and not merely a bother to the teacher. The abnormal child demands sympathetic and careful handling, but he rarely demands exclusion from the salutary discipline of school life.

